# Room-temperature local ferromagnetism and its nanoscale expansion in the ferromagnetic semiconductor Ge_1–*x*_Fe_*x*_

**DOI:** 10.1038/srep23295

**Published:** 2016-03-21

**Authors:** Yuki K. Wakabayashi, Shoya Sakamoto, Yuki-haru Takeda, Keisuke Ishigami, Yukio Takahashi, Yuji Saitoh, Hiroshi Yamagami, Atsushi Fujimori, Masaaki Tanaka, Shinobu Ohya

**Affiliations:** 1 Department of Electrical Engineering and Information Systems, The University of Tokyo, 7-3-1 Hongo, Bunkyo-ku, Tokyo 113-8656, Japan; 2 Department of Physics, The University of Tokyo, Bunkyo-ku, Tokyo 113-0033, Japan; 3 Quantum Beam Science Center, JAEA, Sayo, Hyogo 679-5148, Japan; 4 Department of Physics, Kyoto Sangyo University, Motoyama, Kamigamo, Kita-Ku, Kyoto 603-8555, Japan

## Abstract

We investigate the local electronic structure and magnetic properties of the group-IV-based ferromagnetic semiconductor, Ge_1−*x*_Fe_*x*_ (GeFe), using soft X-ray magnetic circular dichroism. Our results show that the doped Fe 3*d* electrons are strongly hybridized with the Ge 4*p* states, and have a large orbital magnetic moment relative to the spin magnetic moment; i.e., *m*_orb_/*m*_spin_ ≈ 0.1. We find that nanoscale local ferromagnetic regions, which are formed through ferromagnetic exchange interactions in the high-Fe-content regions of the GeFe films, exist even at room temperature, well above the Curie temperature of 20–100 K. We observe the intriguing nanoscale expansion of the local ferromagnetic regions with decreasing temperature, followed by a transition of the entire film into a ferromagnetic state at the Curie temperature.

A major issue that must be addressed for the realization of semiconductor spintronic devices is to achieve room-temperature ferromagnetism in ferromagnetic semiconductors (FMSs) based on the widely used III–V and group-IV materials. In Ga_1−*x*_Mn_*x*_As (GaMnAs), which is the most intensively studied FMS, the highest Curie temperature (*T*_C_) ever reported is 200 K^1^. In GaMnAs, *T*_C_ is limited by the presence of interstitial Mn atoms, which are antiferromagnetically coupled to the substitutional Mn atoms^2^. Recently, however, the group-IV-based FMS, Ge_1–*x*_Fe_*x*_ (GeFe), has been reported to exhibit several attractive features^3^,^4^,^5^. It can be grown epitaxially on Si and Ge substrates without the formation of intermetallic precipitates, and is therefore compatible with mature Si process technology. Unlike GaMnAs, in GeFe, interstitial Fe atoms do not lead to a decrease in *T*_C_^6^, and *T*_C_ can be easily increased to above 200 K by thermal annealing^7^. Furthermore, *T*_C_ does not depend on the carrier concentration, and thus *T*_C_ and resistivity can be controlled separately^8^, which is a unique feature that is only observed in GeFe and is a considerable advantage in overcoming the conductivity mismatch problem between ferromagnetic metals and semiconductors in spin-injection devices. Despite these attractive features, a detailed microscopic understanding of the ferromagnetism in GeFe, which is vitally important for room-temperature applications, is lacking. Here, we investigate the local electronic and magnetic properties of GeFe using X-ray absorption spectroscopy (XAS) and X-ray magnetic circular dichroism (XMCD), which are powerful techniques for element-specific detection of local electronic states and magnetic moments^9^,^10^,^11^,^12^,^13^. We find that nanoscale local ferromagnetic regions remain in the GeFe films even at room temperature, i.e., well above *T*_C_; it follows that GeFe potentially has strong ferromagnetism, which persists even at room temperature. Furthermore, we observe the intriguing feature that ferromagnetic regions, which are formed above *T*_C_ via the ferromagnetic exchange interaction in high-Fe concentration regions of the films, develop and expand as the temperature decreases, and that all of them coalesce at temperatures below *T*_C_. Such a nanoscale expansion of the ferromagnetic regions is a key feature in understanding materials that exhibit single-phase ferromagnetism despite the inhomogeneous distribution of magnetic atoms in the film^6^,^7^,^14^,^15^.

## Results and Discussion

### Basic properties of our GeFe films

**Figure 1:**
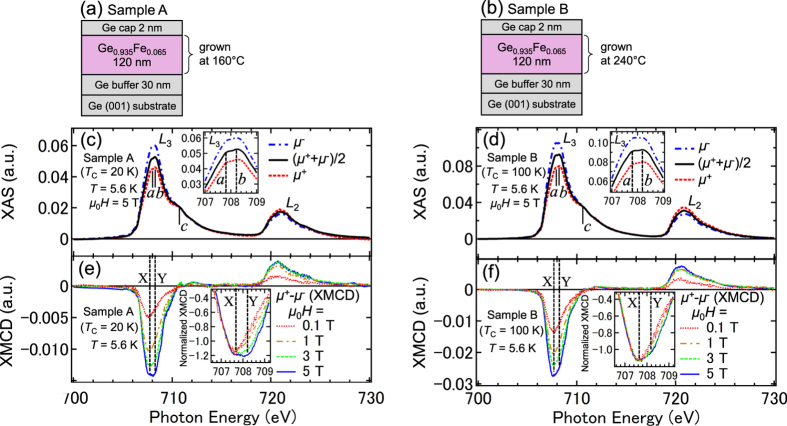
Sample structures, XAS spectra and XMCD spectra. (**a**,**b**) Schematic structures of sample A (**a**) and sample B (**b**). (**c**,**d**) XAS spectra of *μ*^−^, *μ*^+^ and (*μ*^+^ + *μ*^−^)/2 at the *L*_2_ (~721 eV) and *L*_3_ (~708 eV) absorption edges of Fe for sample A (**c**) and sample B (**d**) measured at 5.6 K with *μ*_0_*H* = 5 T applied perpendicular to the film surface. The insets show a magnified plot of the spectra at the Fe *L*_3_ edge. (**e**,**f**) XMCD (=*μ*^+^ − *μ*^−^) spectra at the *L*_2_ and *L*_3_ absorption edges of Fe for sample A (**e**) and sample B (**f**) measured at 5.6 K with various *H* applied perpendicular to the film surface. The insets show a magnified plot of the spectra at the Fe *L*_3_ edge, in which the XMCD data are normalized to 707.3 eV.

We carried out XMCD measurements on two samples (labeled A and B) consisting of a 120-nm-thick Ge_0.935_Fe_0.065_ layer grown on a Ge(001) substrate by low-temperature molecular beam epitaxy (LT-MBE) [Fig. 1(a,b)] (see Methods). The Ge_0.935_Fe_0.065_ layer of sample A was grown at 160 °C, whereas that of sample B was grown at 240 °C. These samples are the same as those studied in ref. 6. From the Arrott plots of the *H* dependence of the magnetic circular dichroism (MCD) measured with visible light with a photon energy of 2.3 eV (corresponding to the *L*-point energy gap of bulk Ge), we found *T*_C_ = 20 K and 100 K for samples A and B, respectively. Detailed crystallographic analyses, including *in situ* reflection high-energy electron diffraction (RHEED), high-resolution transmission electron microscopy (TEM), spatially resolved transmission-electron diffraction (TED) combined with energy-dispersive X-ray spectroscopy (EDX) and X-ray diffraction (XRD), showed that the GeFe films have a diamond-type single-crystal structure without any ferromagnetic precipitates and with nanoscale spatial Fe concentration fluctuations of 4–7% (sample A) and 3–10% (sample B)^6^. We found that *T*_C_ is higher when the fluctuations in the Fe concentration are larger^6^. In addition, channeling Rutherford backscattering (c-RBS) and channeling particle-induced X-ray emission (c-PIXE) measurements showed that ~85% (~15%) of the doped Fe atoms exist at the substitutional (tetrahedral interstitial) sites in both samples A and B, and that the interstitial Fe concentration is not related to *T*_C_^6^. This also indicates that there are *no* ferromagnetic precipitates with different crystal structures in our films.

### XAS and XMCD measurements

We measured the Fe *L*_2,3_-edge XAS spectra [*μ*^+^, *μ*^−^ and (*μ*^+^ + *μ*^−^)/2] of samples A [Fig. 1(c)] and B [Fig. 1(d)] at 5.6 K with *μ*_0_*H* = 5 T applied perpendicular to the film surface. Here, *μ*^+^ and *μ*^−^ refer to the absorption coefficients for photon helicity parallel and antiparallel to the Fe 3*d* majority spin direction, respectively. In both films, three peaks *a*, *b* and *c* are observed at the Fe *L*_3_ edge in the XAS spectra [see also the insets in Fig. 1(c,d)]. We found that the small peak *c* was suppressed by etching the surface with dilute HF, indicating that this peak, which can be assigned to the Fe^3+^ state, originates from a small quantity of surface Fe oxide^16^, which remains even after surface cleaning. Meanwhile, peaks *a* and *b* are assigned to the Fe atoms in GeFe. Peaks *a* and *b* can be assigned to the Fe^2+^ state^17^,^18^.

We measured the Fe *L*_2,3_-edge XMCD (=*μ*^+^ − *μ*^−^) spectra of samples A [Fig. 1(e)] and B [Fig. 1(f)] at 5.6 K with various *H* applied perpendicular to the film surface. Here, we discuss the XMCD intensities at 707.66 eV (X) and 708.2 eV (Y), which correspond to the photon energies of peaks *a* and *b* in the XAS spectra, respectively. When normalized to 707.3 eV, the XMCD spectra with various *H* differ, and the intensity at X grows faster than that at Y as *H* increases, as shown in the insets of Fig. 1(e,f). As shown in Fig. 1(c,d), the shapes of the XAS spectra at the Fe *L*_3_ edge are similar between samples A and B, which have almost the same interstitial Fe concentrations (i.e., 15% of the total Fe content^6^); therefore, we can assign the XMCD intensity at X to the substitutional Fe atoms and the paramagnetic component of the XMCD intensity at Y to the interstitial Fe atoms. We do not observe fine structures due to multiplet splitting at the Fe *L*_3_ edge in both samples, which would be observed if the 3*d* electrons were localized and were not strongly hybridized with other orbitals^19^. These observations indicate that the Fe 3*d* electrons are strongly hybridized with the Ge 4*p* states^20^.

### Determination and analyses of the orbital and spin magnetic moments

**Figure 2:**
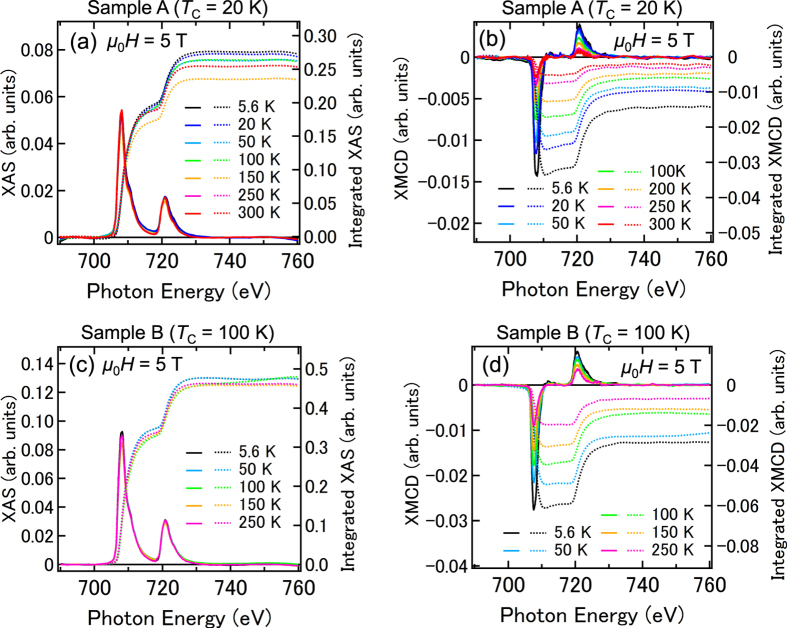
Integrated XAS and XMCD spectra. (**a**,**c**) XAS [=(*μ*^+^ + *μ*^−^)/2] spectra (solid curves) and the XAS signals integrated from 690 eV (dashed curves) of sample A (**a**) and sample B (**c**). (**b**,**d**) XMCD (=*μ*^+^ − *μ*^−^) spectra (solid curves) and the XMCD signals integrated from 690 eV (dashed curves) of sample A (**b**) and sample B (**d**). These measurements were carried out with a magnetic field *μ*_0_*H* = 5 T applied perpendicular to the film surface at 5.6 K (black curves), 20 K (blue curves), 50 K (light blue curves), 100 K (green curves), 150 K (orange curves), 250 K (pink curves), and 300 K (red curves).

We determine the orbital magnetic moment, *m*_orb_, and the spin magnetic moment, *m*_spin_, the orbital magnetic moment relative to the spin magnetic moment, *m*_orb_/*m*_spin_, and the total magnetic moment, *M* = *m*_spin_ + *m*_orb_, of the *substitutional* Fe atoms in accordance with the well-established procedure using the XMCD sum rules^21^,^22^,^23^,^24^,^25^. Figure 2(a) shows the XAS spectra (solid curves) and the XAS signals integrated from 690 eV (dashed curves) of sample A. Figure 2(b) shows the XMCD spectra (solid curves) and the XMCD signals integrated from 690 eV (dashed curves) of sample A. Here, the measurements were carried out with a magnetic field *μ*_0_*H* = 5 T applied perpendicular to the film surface at various temperatures. Figure 2(c,d) shows the same data measured for sample B. For the XMCD sum-rules analyses, we define *r*, *p* and *q* as the following equations at each temperature.


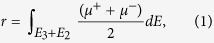



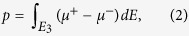



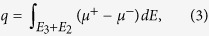


where *E*_3_ (690–718 eV) and *E*_2_ (718–760 eV) represent the integration energy ranges for the *L*_3_ and *L*_2_ absorption edges, respectively. Here, *E* represents the incident photon energy. We can obtain *m*_spin_ and *m*_orb_ of substitutional Fe atoms using the XMCD sum rules, which are expressed as follows:


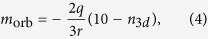



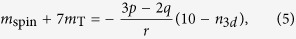


where *n*_3*d*_ and *m*_T_ are the number of 3*d* electrons on the Fe atom and the expectation value of the intra-atomic magnetic dipole operator, respectively. We neglect *m*_T_ because it is negligibly small for Fe atoms at the *T*_d_ symmetry site^24^. By dividing equation (4) by equation (5), *m*_orb_/*m*_spin_ is expressed by


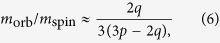


Thus, we can obtain *m*_orb_/*m*_spin_ directly from the XMCD spectra without any assumptions.

**Figure 3:**
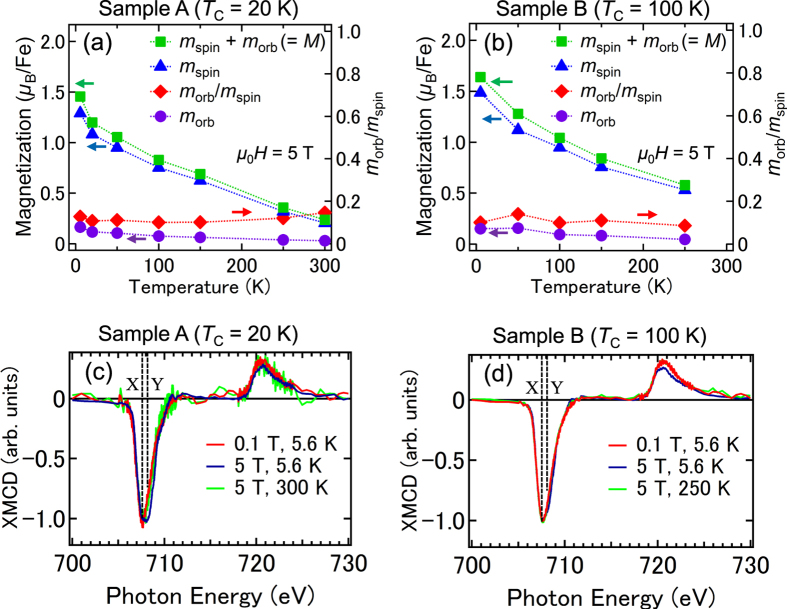
Temperature dependence of *m*_spin_ + *m*_orb_, *m*_spin_, *m*_orb_ and *m*_orb_/*m*_spin_, and the normalized XMCD spectra with different magnetic fields and temperatures. (**a**,**b**) The temperature dependence of *m*_spin_ + *m*_orb_, *m*_spin_, *m*_orb_, and *m*_orb_/*m*_spin_ for sample A (**a**) and sample B (**b**) with an applied magnetic field of *μ*_0_*H* = 5 T. (**c**,**d**) XMCD spectra of samples A (**c**) and B (**d**) normalized to 707.3 eV measured at 5.6 and 300 K with magnetic fields of 0.1 and 5 T applied perpendicular to the film surface.

By the above calculations with equations (2), (3) and (6) using the temperature dependence of XMCD spectra shown in Fig. 2, we obtained the temperature dependence of *m*_orb_/*m*_spin_ of substitutional Fe atoms as shown in Fig. 3(a,b). For sample A, *m*_orb_/*m*_spin_ = 0.12 ± 0.02, and for sample B, *m*_orb_/*m*_spin_ = 0.11 ± 0.03, both of which are positive and larger than that of bulk Fe (where *m*_orb_/*m*_spin_ ~ 0.043^19^); the orbital angular momentum in GeFe is not quenched. The observation that the spin and orbital angular momentum are parallel suggests that the Fe 3*d* shell is more than half filled. This implies that the Fe atoms are in the 2^+^ state rather than in the 3^+^ state, in which the Fe 3*d* shell is half-filled and the orbital angular momentum vanishes. This result is consistent with the peak positions of the XAS spectra (see Fig. 1(c,d)). The large *m*_orb_ is a characteristic property of GeFe, and excludes the possibility of the existence of ferromagnetic Fe metal precipitates in our films.

We describe the derivation of *m*_spin_ and *m*_orb_ using equations (4) and (5). Figure 3(c,d) shows the XMCD spectra of samples A (c) and B (d) normalized to 707.3 eV measured at 5.6 and 300 K with magnetic fields of 0.1 and 5 T applied perpendicular to the film surface. In both films, all the line shapes of the XMCD spectra are almost the same, which means that the paramagnetic component observed at Y in Fig. 1(e,f) is negligibly small in comparison with the entire XMCD spectra and almost all XMCD intensities are composed of the absorptions by the substitutional Fe atoms observed at X in Fig. 1(e,f). This result means that the integrated values of the XMCD spectra *p* [equation (2)] and *q* [equation (3)] can be attributed only to the substitutional Fe atoms. Meanwhile, because the XAS signals have both contributions of the substitutional and interstitial Fe atoms, we reduced the integrated XAS intensity *r* [equation (1)] to 85% of its raw value (85% is the approximate ratio of the substitutional Fe atoms to that of the total Fe atoms in both samples A and B^6^) when using the XMCD sum rules. We note that this assumption, that each substitutional Fe atom and each interstitial Fe atom contribute equally to the integrated XAS intensity [*r* value (equation (1))], does not affect our main conclusions in this paper (see Supplementary Discussion S1). We took *n*_*3d*_ to be 6 and the correction factor for *m*_spin_ to be 0.88^25^ for Fe^2+^ in equations (4) and (5). By the above calculations using the temperature dependence of XAS and XMCD spectra shown in Fig. 2, we obtained the temperature dependence of *m*_spin_, *m*_orb_ and *m*_spin_ + *m*_orb_ (=*M*) of substitutional Fe atoms shown in Fig. 3(a,b). The *M* values obtained by the XMCD measurements are 1.00 *μ*_B_/Fe for sample A and 1.43 *μ*_B_/Fe for sample B when a magnetic field *μ*_0_*H* = 1 T is applied perpendicular to the film surface at 5.6 K. The magnetizations measured by superconducting quantum interference device (SQUID) under the same condition at 5 K are 0.7 *μ*_B_/Fe for sample A and 1.3 *μ*_B_/Fe for sample B^6^. These values are close to those obtained by XMCD. The slight differences may originate from the unavoidable inaccuracy of the subtracting procedure of the large diamagnetic response of the substrate in the SQUID measurements. We see that both *m*_spin_ and *m*_orb_ (and therefore the total magnetic moment *M* = *m*_spin_ + *m*_orb_) are larger in sample B (*T*_C_ = 100 K) than in sample A (*T*_C_ = 20 K) over the entire temperature region when *μ*_0_*H* = 5 T.

**Figure 4:**
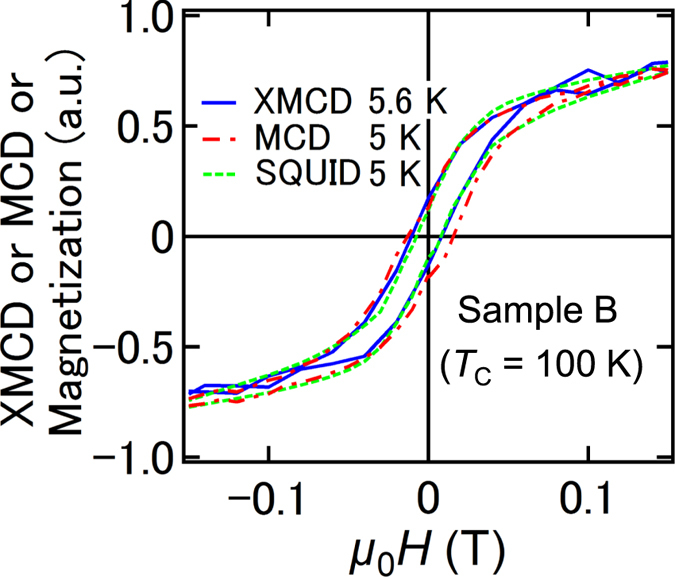
Magnetic field dependence of the normalized XMCD, MCD and magnetization. The *H* dependence of the XMCD intensity at X shown in Fig. 1 (707.66 eV) at 5.6 K, the MCD intensity at 5 K with a photon energy of 2.3 eV corresponding to the *L*-point energy gap of bulk Ge, and the magnetization measured using a SQUID at 5 K for sample B.

Figure 4 shows the *H* dependence of the XMCD intensity at energy X and a temperature of 5.6 K, the MCD intensity measured with visible light of 2.3 eV at 5 K, and the magnetization measured using a SQUID at 5 K for sample B. The shapes of these curves show excellent agreement with each other; it follows that the spin splitting of the valence band composed of the Ge 4*p* orbitals is induced by the Fe 3*d* magnetic moment, which originates from the substitutional Fe atoms, through the *p*–*d* hybridization. The MCD hysteresis curve did not depend on the sweeping speed of the magnetic field unlike superparamagnetic materials with spin blocking [see Supplementary Discussion S2 26,27]. This result supports our understanding that the GeFe films are ferromagnetic below *T*_C_.

### Room-temperature local ferromagnetism and the nanoscale expansion of the local ferromagnetic regions in the GeFe films

**Figure 5:**
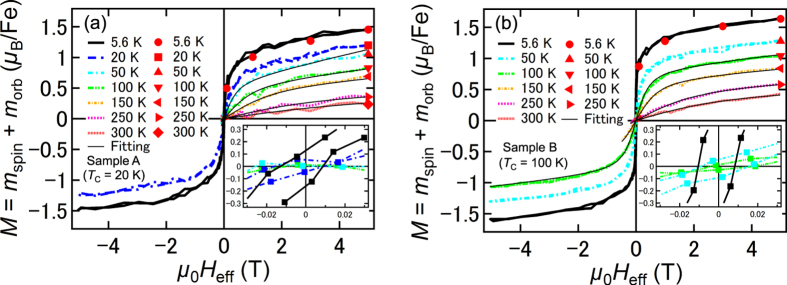
Effective magnetic field dependence of the total magnetic moment. (**a**,**b**) The dependence of the XMCD intensity measured at X on the effective magnetic field *H*_eff_ for sample A (**a**) and sample B (**b**) at various temperatures. The total magnetization (*M* = *m*_spin_ + *m*_orb_) obtained using the XMCD sum rules is also plotted (filled red symbols). We scaled the vertical axis of the XMCD intensity so that it represents *M* at each temperature. In all measurements, *H* was applied perpendicular to the film surface.

Figure 5(a,b) shows the effective magnetic-field (*H*_eff_) dependence of the XMCD intensity measured at X for samples A (a) and B (b) at various temperatures. Here, *M* is also plotted (filled red symbols), and *μ*_0_*H*_eff_ is obtained by subtracting the product of *M* and the density of the substitutional Fe atoms from *μ*_0_*H* to eliminate the influence of the demagnetization field (see Supplementary Discussion S3). The insets show clear hysteresis below *T*_C_ in both samples. The XMCD–*H*_eff_ curves show large curvature above *T*_C_ in both samples [see the main panels of Fig. 5(a,b)], indicating that part of the film is superparamagnetic (SPM) above *T*_C_. It indicates that local ferromagnetic regions form in nanoscale high-Fe concentration regions at temperatures above *T*_C_, and thus *M* can be described by


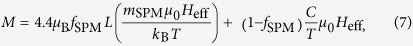


where *f*_SPM_ and *m*_SPM_ are fitting parameters expressing the fraction of SPM substitutional Fe atoms and the magnetic moment per local ferromagnetic region, respectively. Also, *C* is the Curie constant per substitutional Fe atom, and *L* is the Langevin function. Here, 4.4 *μ*_B_ is the *ideal* saturated value of *M*; i.e., *M* = *m*_spin_ + (*m*_orb_/*m*_spin_) ×*m*_spin_, where *m*_spin_ = 4 *μ*_B_ (for Fe^2+^) and *m*_orb_/*m*_spin_ ≈ 0.1 [Fig. 3(a,b)] when all the substitutional Fe atoms are magnetically active. Here, the Curie constant per substitutional Fe atom is obtained using the following equations:


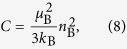



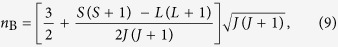


where *μ*_*B*_, *k*_*B*_, *n*_*B*_, *S*, *L* and *J* represent the Bohr magneton, the Boltzmann constant, the effective Bohr magneton number, the spin angular momentum, the orbital angular momentum and the total angular momentum, respectively. Here, *S* = 2 (for Fe^2+^), and *L* = 0.4 (*L* = 2 *S* × *m*_orb_/*m*_spin_, where *m*_orb_/*m*_spin_ ≈ 0.1 as shown in Fig. 3(a,b)), and *J* = 2.4 (=*L* + *S* because the spin and orbital angular momenta of a substitutional Fe atom are parallel) in equation (9). Thus, *n*_*B*_ is estimated to be 5.24. The first and second terms in equation (7) correspond to the SPM and paramagnetic components, respectively. In Fig. 5(a,b), the thin black solid curves correspond to the best fit obtained with equation (7). For sample B, the *M*–*H*_eff_ curves at temperatures in the range 100–300 K are well reproduced by equation (7), which indicates that the ferromagnetic – SPM transition occurs at *T*_C_ = 100 K. With sample A, the *M*–*H*_eff_ curves at temperatures above *T*_C_ (i.e., *T* ≥ 20 K) are well reproduced by equation (7), except for *T* = 20 K, which is probably due to the onset of ferromagnetism. These good fits up to room temperature indicate that ferromagnetic interactions within the nanoscale high-Fe concentration regions remain at room temperature in both samples.

**Figure 6:**
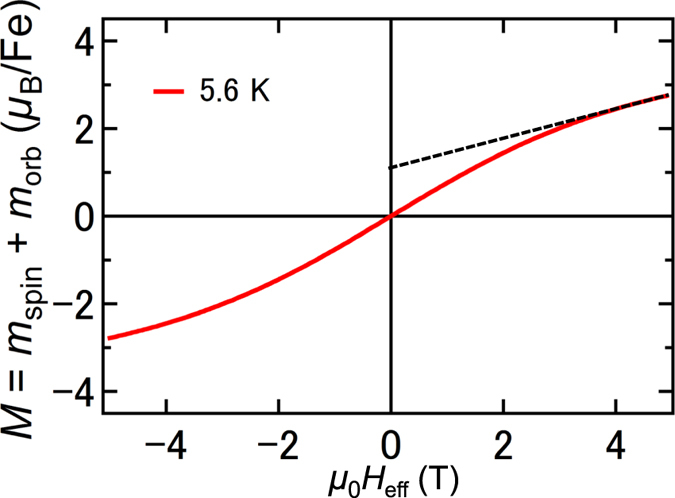
Effective magnetic-field dependence of the total magnetization *M*(=*m*_spin_ + *m*_orb_) per one substitutional paramagnetic Fe at 5.6 K obtained using equation (7).

Here, we estimate the ratios of the substitutional ferromagnetic, paramagnetic, and magnetically inactive Fe atoms to the total number of substitutional Fe atoms at 5.6 K in samples A and B. In this discussion, we only consider substitutional Fe atoms. The obtained results are summarized in Table 1. At 5.6 K, in principle, the *H*_eff_ dependence of *M* (=*m*_spin_ + *m*_orb_) of one substitutional *paramagnetic* Fe atom is expressed by the Langevin function. Thus, theoretically, the *H*_eff_ dependence of *M* of one substitutional paramagnetic Fe atom at 5.6 K is obtained by substituting 4.4 *μ*_B_, 1 and 5.6 K in *m*_SPM_, *f*_SPM_ and *T* of equation (7), respectively (Fig. 6). Here, the experimental *M*–*H*_eff_ curves at 5.6 K shown in Fig. 5(a,b) can be approximately expressed by the sum of the square hysteresis curve originating from substitutional ferromagnetic Fe atoms and the Langevin function originating from substitutional paramagnetic Fe atoms. From the high-field magnetic susceptibility 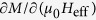
(*μ*_B_/T per Fe) of the *M*–*H*_eff_ curve, we can estimate the fraction of the substitutional paramagnetic Fe atoms. We approximated 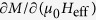 by the slope of the *M*–*H*_eff_ line from 4 T to 5 T. In this way, from Fig. 6, the theoretical 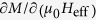 value is estimated to be 0.33 *μ*_B_/T per one substitutional paramagnetic Fe (slope of the black dashed line in Fig. 6). As shown in Fig. 5(a,b), the experimental 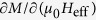 values at 5.6 K in samples A and B are 0.08 *μ*_B_/T and 0.06 *μ*_B_/T, respectively; it follows that the ratios of the substitutional paramagnetic Fe atoms to the total number of substitutional Fe atoms are ~24% (=0.08/0.33) in sample A and ~18% (=0.06/0.33) in sample B. Next, we estimate the fraction of substitutional ferromagnetic Fe atoms. The extrapolated *M* value from the high magnetic field region to *H*_eff_ = 0 in Fig. 5(a,b) is 1.1 *μ*_B_ per Fe atom in sample A, and 1.3 *μ*_B_ per Fe atom in sample B at 5.6 K. Because the *M*–*H*_eff_ curve of the substitutional paramagnetic Fe atoms is expressed by the Langevin function at this temperature as mentioned above, these extrapolated *M* values include a contribution of the substitutional paramagnetic Fe atoms, which is estimated by a linear extrapolation of *M* to *H*_eff_ = 0 in the *M*–*H*_eff_ curve of the substitutional paramagnetic Fe atoms. In Fig. 6, for one substitutional paramagnetic Fe atom, it is 1.1 *μ*_B_ per Fe. Thus, the contribution of the substitutional paramagnetic Fe atoms to the extrapolated *M* value is experimentally ~0.26 *μ*_B_ (=1.1 *μ*_B_ × 0.24) per Fe for sample A and ~0.20 *μ*_B_ (=1.1 *μ*_B_ × 0.18) per Fe for sample B. These results suggest that only ~19% [=(1.1 − 0.26)/4.4] and ~25% [=(1.3 − 0.20)/4.4] of the substitutional Fe atoms are ferromagnetic in samples A and B, respectively. This result means that 57% (=100 − 24 − 19 for sample A and 100 − 18 − 25 for sample B) of the substitutional Fe atoms do not contribute to the total magnetization. We think that some fraction of these substitutional magnetically inactive Fe atoms couple antiferromagnetically. This is also supported by the weak spin-glass behaviour observed in GeFe at very low temperatures^7^.

**Figure 7:**
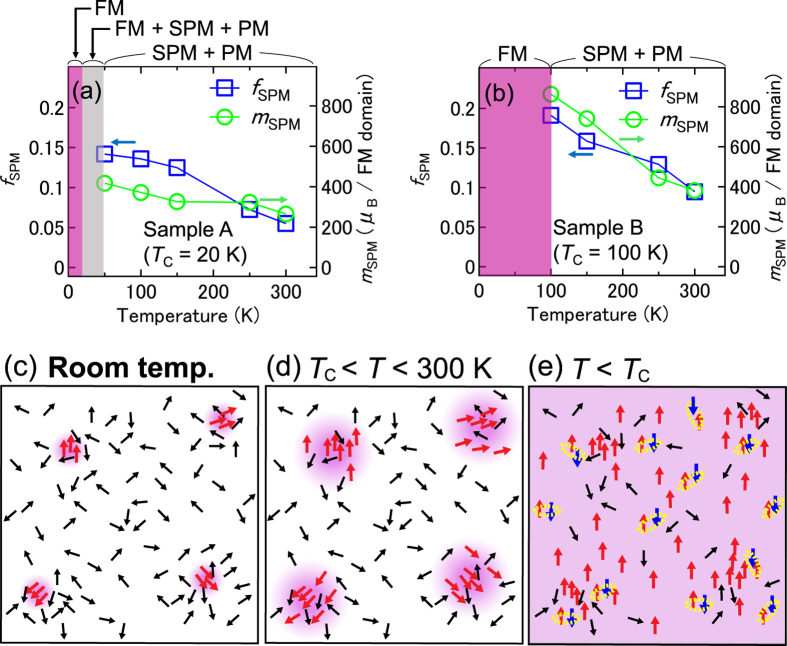
Nanoscale expansion process of the local ferromagnetic regions. (**a**,**b**) The temperature dependence of the best-fit parameters *f*_SPM_ and *m*_SPM_ obtained for sample A (**a**) and sample B (**b**). The red, grey, and white areas indicate ferromagnetic (FM), FM + SPM + paramagnetic (PM), and SPM + PM regions, respectively. (**c**–**e**) Schematic diagrams showing the most likely picture of the magnetic states in the Ge_0.935_Fe_0.065_ films with zero magnetic field at room temperature (i.e., *T* = 300 K) (**c**), *T*_C_ < *T* < 300 K (**d**) and *T* < *T*_C_ (**e**). The small black, red and blue arrows correspond to the magnetic moments of the paramagnetic, ferromagnetic, and antiferromagnetically coupled substitutional Fe atoms, respectively. The red areas indicate ferromagnetic regions. Antiferromagnetically coupled Fe atoms are thought to exist all over the film at temperatures below *T*_C_.

We see a similar trend in the temperature dependence of the fitting parameters *f*_SPM_ and *m*_SPM_ in both films; i.e., *f*_SPM_ and *m*_SPM_ both increase with decreasing temperature (Fig. 7(a,b)). This result implies that the ferromagnetic regions, which form only in the nanoscale high-Fe concentration regions at room temperature [Fig. 7(c)], expand toward lower Fe concentration regions with decreasing temperature [Fig. 7(d)], and finally the entire film becomes ferromagnetic at *T*_C_ [Fig. 7(e)]. This appears to be a characteristic feature of materials that exhibit single-phase ferromagnetism, despite the inhomogeneous distribution of magnetic atoms in the film^6^,^7^. As shown in Fig. 7(a,b), *f*_SPM_ and *m*_SPM_ are larger in sample B than in sample A, which can be attributed to the difference in spatial fluctuations of the Fe concentration, which are 4–7% in sample A and 3–10% in sample B^6^. The larger the nonuniformity of the Fe distribution is, the larger each local ferromagnetic region, *f*_SPM_, and *m*_SPM_ become, and the local ferromagnetic regions can be more easily connected magnetically, resulting in a higher *T*_C_.

### Summary

We have investigated the local electronic structure and magnetic properties of the doped Fe atoms in the Ge_0.935_Fe_0.065_ films, which have a diamond-type single-crystal structure without any ferromagnetic precipitates and with nanoscale spatial Fe concentration fluctuations, using XAS and XMCD. The Fe atoms appear in the 2^+^ state, with the 3*d* electrons strongly hybridized with the 4*p* electrons in Ge; this results in a delocalized 3*d* nature and long-range ferromagnetic ordering, leading to the excellent agreement between the *H* dependence of magnetization, MCD and XMCD. Using the XMCD sum rules, we obtained the *M*–*H*_eff_ curves, which can be explained by the coexistence of SPM and paramagnetic properties at temperatures above *T*_C_. The fitting results clearly show that the local ferromagnetic regions, which exist at room temperature, expand with decreasing temperature, leading to a ferromagnetic transition of the entire system at *T*_C_. The nonuniformity of the Fe concentration seems to play a crucial role for the formation of the ferromagnetic regions, and our results indicate that strong ferromagnetism is inherent to GeFe, and persists at room temperature. Such a nanoscale expansion of the ferromagnetic regions is a key feature in understanding materials that exhibit single-phase ferromagnetism (i.e., where the film is free from any ferromagnetic precipitates) despite the *inhomogeneous* distribution of magnetic atoms in the film^6^,^7^,^14^,^15^.

## Methods

### Sample preparation

The Ge_0.935_Fe_0.065_ thin films were grown on Ge(001) substrates by LT-MBE. The growth process is described as follows. After the Ge(001) substrate was chemically cleaned and its surface was hydrogen-terminated by buffered HF solution, it was introduced in the MBE growth chamber through an oil-free load-lock system. After degassing the substrate at 400 °C for 30 minutes and successive thermal cleaning at 900 °C for 15 min, we grew a 30-nm-thick Ge buffer layer at 200 °C, which was followed by the growth of a 120-nm-thick Ge_0.935_Fe_0.065_ layer at *T*_S_ = 160 (sample A) or 240 °C (sample B). After that, we grew a 2-nm-thick Ge capping layer at 200 °C to avoid the surface oxidation of the GeFe layer. The *in situ* RHEED was used to check the crystallinity and morphology of the surface during the growth. The diffraction pattern of the Ge_0.0935_Fe_0.065_ showed intense and sharp 2 × 2 streaks with no extra spots, which indicate a 2-dimensional growth mode and exhibit a diamond-type single-crystal structure. To remove the oxidized surface layer, the samples were briefly etched in dilute hydrofluoric acid (HF) prior to loading into the XAS (XMCD) vacuum chamber.

### XAS and XMCD measurements

We performed XAS and XMCD measurements at the soft X-ray beamline BL23SU of SPring-8 with a twin-helical undulator of in-vacuum type^28^. The monochromator resolution was *E*/Δ*E* > 10000. The XAS and XMCD spectra were obtained by reversing photon helicity at each energy point and were recorded in the total-electron-yield mode. The XMCD spectra were taken both for positive and negative applied magnetic fields and were averaged in order to eliminate experimental artifacts. Backgrounds of the XAS spectra at the Fe *L*_2,3_-edge were assumed to be hyperbolic tangent functions.

## Additional Information

**How to cite this article**: Wakabayashi, Y. K. *et al.* Room-temperature local ferromagnetism and its nanoscale growth in the ferromagnetic semiconductor Ge_1–*x*_Fe_*x*_. *Sci. Rep.* **6**, 23295; doi: 10.1038/srep23295 (2016).

## Supplementary Material

Supplementary Information

## Figures and Tables

**Table 1:** Ratios of the substitutional ferromagnetic, paramagnetic, and magnetically inactive Fe atoms to the total number of substitutional Fe atoms at 5.6 K in samples A and B.

Sample	Ferromagnetic	Paramagnetic	Inactive
A	19%	24%	57%
B	25%	18%	57%
