# Supplementary material for: Room-temperature local ferromagnetism and its nanoscale expansion in the ferromagnetic semiconductor Ge1–xFex
Source: Sci Rep. 2016 Mar 21;6:23295. doi: 10.1038/srep23295 (PMC4800415; doi:10.1038/srep23295)
Supplement: Supplementary Information [file srep23295-s1.pdf]

Supplemental Information for Room-temperature local ferromagnetism and its nanoscale expansion in the ferromagnetic semiconductor $Ge_{1-x}Fe_x$

Yuki K. Wakabayashi,[1] Shoya Sakamoto,[2] Yuki-haru Takeda,[3] Keisuke Ishigami,[2] Yukio Takahashi,[2] Yuji Saitoh,[3] Hiroshi Yamagami,[3] Atsushi Fujimori,[2] Masaaki Tanaka,[1] and Shinobu Ohya[1]

[1]*Department of Electrical Engineering and Information Systems, The University of Tokyo, 7-3-1 Hongo, Bunkyo-ku, Tokyo 113-8656, Japan*
[2]*Department of Physics, The University of Tokyo, Bunkyo-ku, Tokyo 113-0033, Japan*
[3]*Synchrotron Radiation Research Unit, JAEA, Sayo, Hyogo 679-5148, Japan*

Discussion S1. The influence of the assumption used in our manuscript on the main conclusions

In our manuscript, we assumed, that each substitutional Fe atom and each interstitial Fe atom, which exist with the ratio of 85% and 15% of the doped Fe atoms, respectively[S1], contribute equally to the integrated XAS intensity expressed by equation (1) in the main text. Hereinafter, we call this assumption as assumption A in this Supplemental Information. This was used for deriving the following main conclusions (hereinafter referred to as Finding I, II and III, respectively, in this Supplemental Information).

I. The first observation of the intriguing expansion of nanoscale local ferromagnetic regions with decreasing temperature, followed by a transition of the entire film into a ferromagnetic state at the Curie temperature as shown in Fig. 7 in the main text.

II. There are Fe atoms antiferromagnetically coupled to the substitutional Fe atoms below T_C.

III. The fraction f_{SPM} of superparamagnetic substitutional Fe atoms and the magnetic moment m_{SPM} per local ferromagnetic region are correlated with T_C and the nonuniformity of the Fe concentration; the larger the nonuniformity of the Fe distribution is, the larger each local ferromagnetic region, f_{SPM}, and m_{SPM} become, and the local ferromagnetic regions can be more easily connected magnetically, resulting in a higher T_C.

Here, we describe that these conclusions do *not* depend on assumption A.

Finding I. The first observation of the intriguing expansion of nanoscale local

ferromagnetic regions with decreasing temperature, followed by a transition of the entire film into a ferromagnetic state at the Curie temperature, as shown in Fig. 7 in the main text.

For obtaining Finding I, we derived the M values from the XAS and XMCD data in accordance with the well-established procedure using the XMCD sum rules[S 2], as described in the main text. For obtaining the M values of the *substitutional* Fe atoms (85% of the total Fe atoms) using equations (1)-(5) in the main text under assumption A, we reduced the r values to 85% of its raw values. Here, we neglected the paramagnetic components observed at Y (708.2 eV), which is assigned to the interstitial Fe atoms, in Figs. 1(e) and 1(f) in the main text, because they are negligibly small for the XMCD sum-rules analyses as described in the main text.

To check how assumption A affects our main conclusion, we calculate the M values based on another assumption that the influence of the interstitial Fe atoms, which exist at a rate of 15% of the doped Fe atoms[S1], on the r values is negligibly small (hereinafter referred to as assumption B in this Supplemental Information). Under assumption B, we calculate the M values using equations (1)-(5) in the main text with the raw r values. Figure S1(a),(b) shows the M-H_{eff} curves with assumption B. These M values are 0.85 times smaller than those shown in Fig. 5(a),(b) of the main text, which are derived under assumption A. Note that the shape of the M-H_{eff} curves is unchanged. In Fig. S1(a),(b), the thin black solid curves correspond to the best fit obtained with equation (7) in the main text. The temperature dependences of the fitting parameters f_{SPM} and m_{SPM} shown in Fig. S2 are uniquely determined from the numerical fittings using equation (7) as shown in Fig. S1(a),(b). We also see the same trend in the temperature dependence of the fitting parameters as that shown in Fig. 7(a),(b), which is derived under assumption A, of the main text; i.e., f_{SPM} and m_{SPM} both increase with decreasing temperature. Thus, we can obtain Finding I regardless of the assumption used in our manuscript. This is because the temperature and sample dependences of the shape of the M-H_{eff} curves do not depend on the assumption.

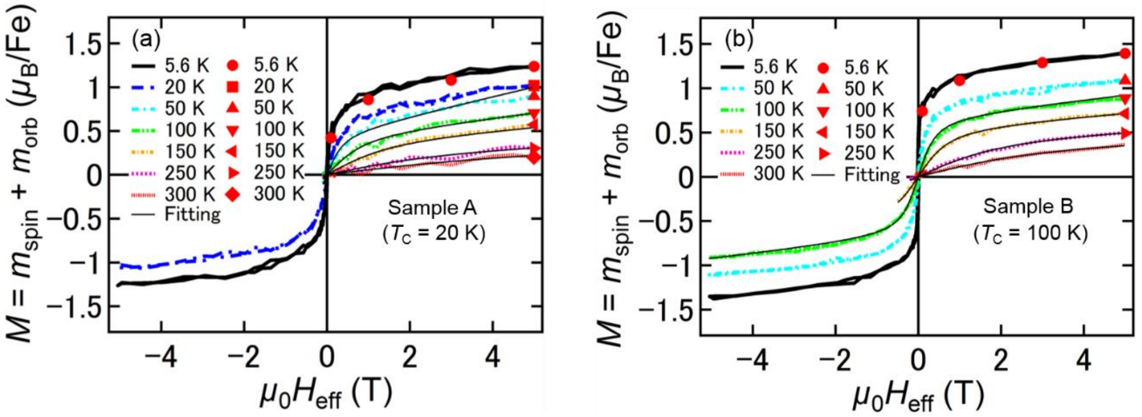

Fig. S1. (a), (b) The dependence of the XMCD intensity measured at X (707.66 eV) on the effective magnetic field H_{eff} for sample A (a) and sample B (b) at various temperatures when we assume that all of the XAS intensity is attributed to the substitutional Fe atoms (assumption B). The total magnetization ($M = m_{spin} + m_{orb}$) obtained using the XMCD sum rules is also plotted (filled red symbols). We scaled the vertical axis of the XMCD intensity so that it represents M at each temperature. In all measurements, H was applied perpendicular to the film surface.

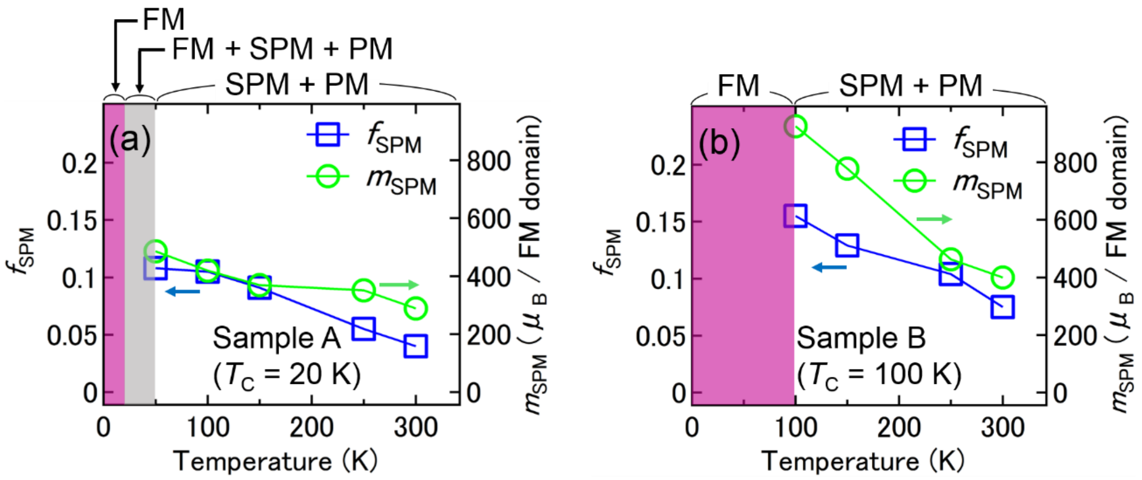

Fig. S2. (a), (b) The temperature dependence of the best-fit parameters f_{SPM} and m_{SPM} obtained for sample A (a) and sample B (b) when we assume that all of the XAS intensity is attributed to the substitutional Fe atoms (assumption B). The red, grey, and white areas indicate ferromagnetic (FM), FM + SPM + paramagnetic (PM), and SPM + PM regions, respectively.

Finding II. There are Fe atoms antiferromagnetically coupled to the substitutianal Fe atoms below T_C.

We derive this conclusion from the extrapolated *M* value and the high-field magnetic susceptibilities $\partial M/\partial(\mu_0 H_{\mathrm{eff}})$ (μ_B/T per Fe atom) at 5.6 K. This conclusion does *not* depend on the assumption used in our manuscript (assumption A), because the assumption has only a slight influence on the *M* values (the shape of the *M*-H_{eff} curves is not changed).

Here, we derive Finding II under assumption B in order to show that Finding II is obtained regardless of the assumption. In Fig. S1(a),(b), the high-field magnetic susceptibilities $\partial M/\partial(\mu_0 H_{\mathrm{eff}})$ in samples A and B at 5.6 K, when assuming that all of the XAS intensity is attributed to the substitutional Fe atoms (assumption B), are 0.07 μ_B/T and 0.05 μ_B/T, respectively. Because, in principle, $\partial M/\partial(\mu_0 H_{\mathrm{eff}})$ of one substitutional paramagnetic Fe atom should be 0.33 μ_B/T as explained in the main text, the ratios of the paramagnetic Fe atoms to the total number of substitutional Fe atoms are only ~21% (= 0.07/0.33) in sample A and ~15% (= 0.05/0.33) in sample B. These values are almost the same as those obtained under assumption A in the main text (24% and 18%). In Fig. S1(a),(b), the extrapolated *M* value, which is obtained from a linear extrapolation of *M* from the high magnetic field region to H_{eff} = 0 at 5.6 K, is 0.94 μ_B per Fe atom in sample A, and 1.11 μ_B per Fe atom in sample B. Because the *M*-H_{eff} curve of the substitutional paramagnetic Fe atoms is expressed by the Langevin function at this temperature, these values include a contribution of substitutional paramagnetic Fe atoms, which is estimated by a linear extrapolation of *M* to H_{eff} = 0 in the *M*-H_{eff} curve of substitutional paramagnetic Fe atoms. In Fig. 6, for one substitutional paramagnetic Fe atom, it is 1.1 μ_B per Fe. Thus, the contribution of the substitutional paramagnetic Fe atoms to the extrapolated *M* value is experimentally ~0.23 μ_B (= 1.1 μ_B $\times$ 0.21) per Fe for sample A and ~0.17 μ_B (= 1.1 μ_B $\times$ 0.15) per Fe for sample B. This result suggests that only ~16% [= (0.94-0.23)/4.4] and ~21% [= (1.11-0.17)/4.4] of the substitutional Fe atoms are ferromagnetic in samples A and B, respectively. These values are almost the same as those obtained under assumption A in the main text (19% and 25%). This means that some fraction of the moment of the Fe atoms is missing, and thus suggests that there are Fe atoms that couple antiferromagnetically with the ferromagnetic Fe atoms in the films below T_C. Thus, we can obtain Finding II regardless of the assumption.

Finding III. The f_{SPM} and m_{SPM} values are correlated with T_C and with the nonuniformity of the Fe concentration; the larger the nonuniformity

of the Fe distribution is, the larger each local ferromagnetic region, f_{SPM}, and m_{SPM} become, and the local ferromagnetic regions can be more easily connected magnetically, resulting in a higher T_C.

This conclusion also does not depend on the assumption used in our manuscript (assumption A), because the temperature and sample dependences of the shape of the M-H_{eff} curves do not depend on the assumption.

Here, we show that we can derive Finding III under assumption B in order to show that Finding III is obtained regardless of the assumption. As shown in Fig. S2, the obtained f_{SPM} and m_{SPM} are larger in sample B (T_C = 100 K) than in sample A (T_C = 20 K) when we assume that all of the XAS intensity is attributed to the substitutional Fe atoms (assumption B). This result can be attributed to the larger Fe concentration fluctuation in sample B than that in sample A observed in Ref. S1. Thus, we can obtain Finding III regardless of the assumption because the temperature and sample dependences of the shape of the M-H_{eff} curves do not depend on the assumption.

Discussion S2. Exclusion of the possibility of spin-blocking below T_C.

Here, we show that our GeFe films are ferromagnetic, but not superparamagnetic below T_C. Figure S3(a),(b) shows the magnetic-field H dependence of the MCD intensity for sample B at 5 K (a) and 50 K (b) with a photon energy of 2.3 eV measured at different sweeping speeds of H (0.0067 – 0.002 T/s) applied perpendicular to the film surface. The shapes of these curves at each temperature show excellent agreement with each other, meaning that the MCD hysteresis curve does not depend on the sweeping speed of H unlike superparamagnetic materials with spin blocking[S3,S4]. From this result, we can exclude the possibility of the spin-blocking with fast relaxation. We can also exclude the spin-blocking with slow relaxation because the clear hysteresis is observed above the blocking (spin-glass transition) temperature ($<$ 26 K) as mentioned below.

Figure S3(c) shows the magnetization versus temperature (M-T) curves of sample B measured by SQUID. In the zero-field-cooling (ZFC) process shown by the blue curve, M was measured with H = 100 Oe applied perpendicular to the film surface with increasing temperature after the sample was cooled down to 4 K from room temperature without a magnetic field. In the field-cooling (FC) process shown by the red curve, M was measured with decreasing temperature from room temperature to 4 K under H = 100 Oe applied perpendicular to the film surface. The inset shows a magnified plot near 0 K. At very low temperature, we see a slight difference between the M values in ZFC and FC

processes, which means that there is a weak spin-glass phase in the film only at very low temperature[S5]. The spin-glass transition temperature T_{SG}(100 Oe), which is defined as the temperature at which the difference between the M values in ZFC and FC processes appears when H is 100 Oe, is 10 K in sample B. As shown in Ref. S5, this value is lower than that of the $Ge_{0.895}Fe_{0.105}$ film grown at 240°C (i.e. T_{SG}(100 Oe) $\approx$ 24 K), which has a spin-glass transition temperature T_{SG}(0 Oe) of ~26 K[S5]. This result means that the T_{SG}(0 Oe) of sample B is lower than 26 K. The T_C value of sample B (100 K) is much higher than T_{SG}(0 Oe) ($<$ 26 K), and more than 80% of M remains in the ZFC process even at 4 K (Fig. S3(c)). Therefore, the ferromagnetism is much stronger than the spin-glass phase and is dominant in the entire temperature region below T_C. In the case of the superparamagnetic particles with slow relaxation, the magnetic hysteresis is only observed below the blocking temperature, as shown in Ref. S3. Thus, we can conclude that the GeFe films are ferromagnetic below T_C.

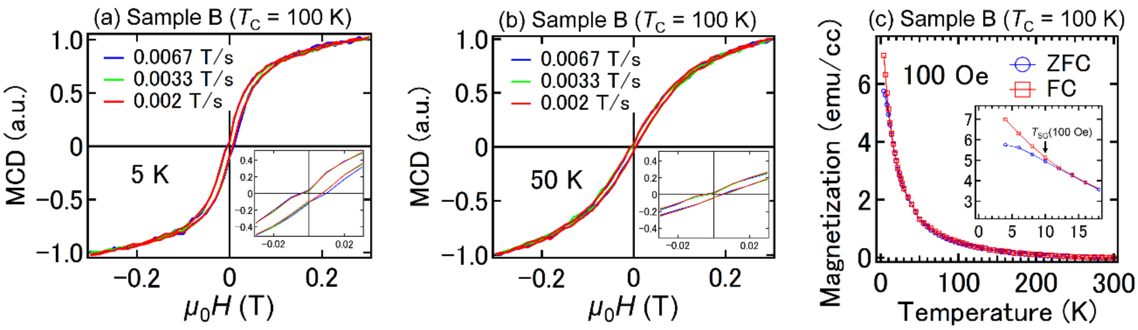

Figure S3. (a), (b) The H dependence of the MCD intensity for sample B at 5 K (a) and 50 K (b) with a photon energy of 2.3 eV measured at different sweeping speeds of H applied perpendicular to the film surface. (c) Magnetization versus temperature (M-T) curves of sample B. The measurements were performed in the two processes of field cooling (FC, red curve) and zero field cooling (ZFC, blue curve) with H = 100 Oe applied perpendicular to the film surface.

Discussion S3. Influence of the demagnetization field

The demagnetization field is not significant in GeFe films. As can be seen in Fig. S4, the magnetization curves are almost the same whether or not we consider the demagnetization field. This is because the magnetic moments of the entire films are small.

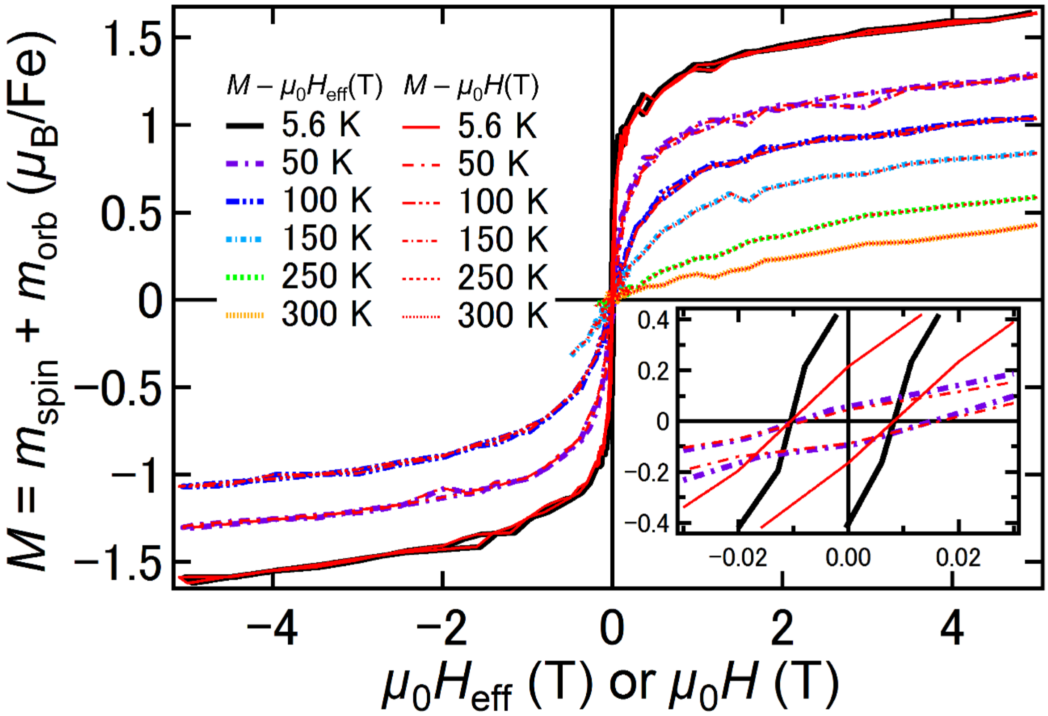

Fig. S4. The dependence of the XMCD intensity measured at X on the effective magnetic field H_{eff} or the magnetic field H (red curves) for sample B at various temperatures. We scaled the vertical axis of the XMCD intensity so that it represents M at each temperature. In all measurements, H was applied perpendicular to the film surface.

References

[S1] Wakabayashi, Y. K., Ohya, S., Ban, Y. & Tanaka, M. Important role of the non-uniform Fe distribution for the ferromagnetism in group-IV-based ferromagnetic semiconductor GeFe. *J. Appl. Phys.* **116**, 173906 (2014).

[S2] Chen, C. T. *et al*. Experimental Confirmation of the X-Ray Magnetic Circular Dichroism Sum Rules for Iron and Cobalt. *Phys. Rev. Lett.* **75**, 152 (1995).

[S3] Park, Y., Adenwalla, S., Felcher, G. P. & Bader, S. D. Superparamagnetic relaxation of Fe deposited on MgO(001). *Phys. Rev. B* **52**, 12779 (1995).

[S4] Chowdary, K. M. & Majetich, S. A. Frequency-dependent magnetic permeability of $Fe_{10}Co_{90}$nanocomposites. *J. Phys. D: Apple. Phys.* **47**, 1750001 (2014).

[S5] Wakabayashi, Y. K., Ban, Y., Ohya, S. & Tanaka, M. Annealing-induced enhancement of ferromagnetism and nanoparticle formation in the ferromagnetic semiconductor GeFe. *Phys. Rev. B* **90**, 205209 (2014).
